# Assessment of predictors of early postoperative complications after primary robotically assisted Roux-en-Y gastric bypass: a multicenter, retrospective cohort study

**DOI:** 10.1007/s00464-022-09766-x

**Published:** 2022-12-09

**Authors:** Pouya Iranmanesh, Shinil K. Shah, Mickael Chevallay, Christian Toso, Stefan P. Mönig, Monika E. Hagen, Erik B. Wilson, Minoa K. Jung

**Affiliations:** 1 Division of Digestive Surgery, Geneva University Hospital and Faculty of Medicine, Geneva, Switzerland; 2 Division of Minimally Invasive and Elective General Surgery, Department of Surgery, McGovern Medical School, University of Texas Health Science Center at Houston, Houston, TX USA

**Keywords:** Roux-en-Y gastric bypass, Robotic surgery, Bariatric surgery

## Abstract

**Background:**

Robotic Roux-en-Y gastric bypass (RRYGB) is performed in an increasing number of bariatric centers worldwide. Previous studies have identified a number of demographic and clinical variables as predictors of postoperative complications after laparoscopic Roux-en-Y gastric bypass (LRYGB). Some authors have suggested better early postoperative outcomes after RRYGB compared to LRYGB. The objective of the present study was to assess potential predictors of early postoperative complications after RRYGB.

**Methods:**

A retrospective analysis of two prospective databases containing patients who underwent RRYGB between 2006 and 2019 at two high volumes, accredited bariatric centers was performed. Primary outcome was rate of 30 day postoperative complications. Relevant demographic, clinical and biological variables were entered in a multivariate, logistic regression analysis to identify potential predictors.

**Results:**

Data of 1276 patients were analyzed, including 958 female and 318 male patients. Rates of overall and severe 30 day complications were 12.5% (160/1276) and 3.9% (50/1276), respectively. Rate of 30 day reoperations was 1.6% (21/1276). The overall gastrointestinal leak rate was 0.2% (3/1276). Among various demographic, clinical and biological variables, male sex and ASA score >2 were significantly correlated with an increased risk of 30 day complication rates on multivariate analysis (OR 1.68 and 1.67, p=0.005 and 0.005, respectively).

**Conclusion:**

This study identified male sex and ASA score >2 as independent predictors of early postoperative complications after RRYGB. These data suggest a potentially different risk profile in terms of early postoperative complications after RRYGB compared to LYRGB. The robotic approach might have a benefit for patients traditionally considered to be at higher risk of complications after LRYGB, such as those with BMI >50. The present study was however not designed to assess this hypothesis and larger, prospective studies are necessary to confirm these results.

Bariatric and metabolic surgery is considered the most effective treatment for obesity and its related comorbidities [1, 2]. Minimally invasive techniques have become the standard approach to perform these procedures, among which Roux-en-Y gastric bypass (RYGB) is one of the most commonly performed worldwide [3]. Results from large cohort studies show overall postoperative complication rates of 15–20% and gastrointestinal leak rates of 0.1–1.2% in patients undergoing primary laparoscopic RYGB (LRYGB) [4–8]. A number of predictors of postoperative complications after laparoscopic RYGB have been identified in previous studies, including among others male sex, higher BMI, presence of comorbidities such as diabetes, smoking and use of anticoagulation [9–11]. Robotically assisted laparoscopic RYGB (RRYGB) is performed in an increasing number of bariatric centers across the world [12]. The robotic platform offers tri-dimensional visualization, increased ergonomics, tremor filtration, articulated instruments and the possibility of performing handsewn anastomoses similarly to open surgery [13, 14]. Despite the absence of randomized data, outcomes after RRYGB seem to be at least equivalent to LRYGB according to several large cohort studies, with some authors suggesting better outcomes in terms of anastomotic leak and strictures rates, especially in patients undergoing revisional bariatric surgery [15–20]. Given the hypotheses that patients undergoing RRYGB might have different patterns of postoperative complications and improved postoperative outcomes compared to LRYGB, the objective of the present study was to identify predictors of early postoperative complications after primary RRYGB.

## Material and methods

### Design, setting and participants

Two high-volume, nationally accredited bariatric centers located in two different countries contributed to the present study: University of Texas Health Science Center at Houston and Memorial Hermann—Texas Medical Center (Houston, TX, USA) and Geneva University Hospital (Geneva, Switzerland). Both centers maintain a prospective database containing data of all patients who undergo bariatric procedures. A retrospective analysis of these two databases was performed.

Inclusion criteria were:

- Patients ≥ 18 years undergoing primary RRYGB between 2006 and 2019
- Minimum postoperative length of follow-up of 1 month

Exclusion criteria were:

- Patients undergoing reoperative RRYGB, defined by revision or reversal of an existing RYGB, or by conversion from another bariatric procedure
- Patients undergoing RRYGB after a previous anti-reflux procedure

### Technique

All surgeries were performed using the Si or Xi version of the da Vinci® Surgical System (Intuitive Incorporation, Sunnyvale, CA, USA). Both institutions performed end-to-side gastrojejunal anastomoses using a fully handsewn technique with absorbable suturing material, in either a single- (Geneva University Hospital) or a double-layer fashion (Texas Medical Center). Jejunojejunal anastomoses were performed in a side-to-side fashion either using a single-layer, fully handsewn technique with absorbable suturing material (Geneva University Hospital) or with a 60 mm endoscopic linear stapler with handsewn enterotomy defects closure (Texas Medical Center). Other details of our operative technique used for RRYGB have been previously published elsewhere [21].

### Collected data

Baseline characteristics included age, sex, body mass index (BMI), preoperative American Society of Anesthesiology (ASA) scores, presence of obesity-related comorbidities such as type 2 diabetes mellitus (T2D), hypertension, dyslipidemia, obstructive sleep apnea syndrome (OSAS) and gastroesophageal reflux disease (GERD), and the following plasma laboratory values: creatinine, ferritin, albumin and hemoglobin. For subsequent binary analyses, patients were divided in the following subgroups according to clinically relevant cut-offs: BMI ≤ or >50 and ASA score ≤ or >2. For laboratory values, abnormal values were defined by >80 µmol/l for creatinine, <30 µg/l for ferritin, <35 g/l for albumin and <120 g/l for hemoglobin, respectively. Primary outcome was the number of early postoperative complications, defined by any complication occurring within 30 days after surgery, and ranked according to the Dindo-Clavien classification [22]. Severe complications were defined by a score ≥IIIa. Secondary outcomes were early reoperations (i.e. within 30 days after surgery), conversions to open surgery and number of gastrointestinal leaks.

### Statistical analysis

All calculations were performed using SPSS Statistics version 25 (IBM Corporation, Armonk, NY). Primary and secondary outcomes were initially analyzed separately for each center to ensure comparability between patient populations. Continuous variables were reported as mean with standard deviation, and Mann–Whitney-Wilcoxon test was used to detect differences. Categorical variables were reported as number and proportion, and Chi-Square or Fisher’s exact test was used where appropriate to detect differences. Clinically pertinent variables were entered into a multivariate, logistic regression model to assess independent predictors of overall and severe postoperative complications. A *p*-value ≤0.05 was considered statistically significant.

### Ethical and quality considerations

Since all data were used anonymously for research purposes exclusively, necessity of patient written consent was waived by the institutional review boards at Geneva University Hospital and the University of Texas Health Science Center at Houston. All patient charts were reviewed manually by the primary author to ensure data completeness and avoid potential collection biases. Obesity-related comorbidities such as T2D, hypertension, dyslipidemia and OSAS were defined according to the American Society for Metabolic and Bariatric Surgery guidelines for standardized reporting [23]. The diagnosis of GERD was based on a history of typical symptoms at Geneva University Hospital and according to the GerdQ questionnaire at University of Texas Health Science Center. Reporting of results was based on the Strengthening the Reporting of Observational Studies in Epidemiology (STROBE) Statement.

## Results

Data of 1276 patients who met inclusion criteria were analyzed. Among them, 811 patients underwent bariatric surgery at Geneva University Hospital between 2006 and 2018 and 465 at Texas Medical Center between 2012 and 2019. There were no exclusion of patients due to insufficient postoperative follow-up (<1 month). Baseline characteristics are shown in Table 1. Mean age, mean BMI, ASA scores and prevalence of obesity-related comorbidities were significantly higher in patients who underwent surgery at Texas Medical Center, except for the prevalence of dyslipidemia which was higher among Geneva University Hospital patients.

**Table 1:** Baseline characteristics

	Overall (*N* = 1276)	Geneva university hospital (*N* = 811)	Texas medical center (N = 465)	*P*-value
Age (mean [±SD], years)	44.4 [±11.4]	43.3 [±10.7]	46.5 [±12.3]	<0.00001*
Female patients (N,%)	958 (75.1%)	599 (73.9%)	359 (77.2%)	0.2014
BMI (mean [±SD], kg/m^2^)	44.6 [±7.1]	43.5 [±5.6]	46.5 [±8.9]	<0.00001*
BMI ≥50 (N,%)	236 (18.5%)	106 (13.1%)	130 (28%)	<0.00001*
ASA score (N,%):				
1	2 (0.2%)	2 (0.2%)	0	0.5364
2	553 (43.3%)	552 (68.1%)	253 (54.4%)	<0.00001*
3	720 (56.4%)	256 (31.6%)	212 (45.6%)	<0.00001*
4	1 (0.1%)	1 (0.1%)	0	
Comorbidities (N,%):				
Hypertension	561 (44.0%)	267 (32.9%)	294 (63.2%)	<0.00001*
T2D	442 (34.6%)	243 (30.0%)	199 (42.8%)	<0.00001*
Dyslipidemia^a^	923 (72.3%)	720 (88.8%)	203 (43.7%)	<0.00001*
GERD^a^	431 (33.8%)	197 (24.3%)	234 (50.3%)	<0.00001*
OSAS	432 (33.9%)	258 (31.8%)	174 (37.4%)	0.0428*

*SD* standard deviation, *BMI* body mass index, *ASA* American society of anesthesiologists, *T2D* type 2 diabetes mellitus, *GERD* gastroesophageal reflux disease, *OSAS* obstructive sleep apnea syndrome

Asterisks indicate statistically significant *p*-values

^a^Diagnostic criteria of dyslipidemia and GERD were different between the two institutions

Primary and secondary outcomes, as well as complications breakdown by grade, are shown in Table 2. There were no statistically significant differences in terms of 30 day postoperative complications (overall and severe), 30 day reoperations, conversions to open surgery and gastrointestinal leaks between the two institutions. Severe complications included (*N*=50, Dindo-Clavien ranking is given (i): six intraluminal bleedings treated endoscopically (IIIa), one early gastrojejunal ulcer diagnosed endoscopically (IIIa), one massive pulmonary embolism requiring thrombolysis (IIIa), five extraluminal bleedings requiring reoperation (IIIb), six early incisional hernias requiring reoperation (IIIb), four early gastrojejunal strictures treated endoscopically (IIIb), six obstructions at the jejunojejunostomy or its afferent limbs requiring reoperation (IIIb), one perforated gastrojejunal ulcer requiring reoperation, one biliary leak of the cystic stump requiring reoperation (IIIb), one gastrojejunal and 1 gastric remnant leak requiring reoperation (IIIb), 16 patients requiring intensive care unit admission (IV) due to respiratory failure (*N*=11), heart failure (*N*=1), massive pulmonary embolus (*N*=1), severe sepsis (*N*=1) and laryngeal edema (*N*=2), and one death due to massive pulmonary and carotid embolism (V).

**Table 2:** Primary and secondary outcomes, with breakdown of complications by grade

	Overall (*N*=1276)
Overall complications (%)	160 (12.5%)
Severe complications (%)	50 (3.9%)
30 day reoperations (%)	21 (1.6%)
Conversions to open surgery (%)	3 (0.2%)
Gastrointestinal leaks (%)	3 (0.2%)
Gastric remnant	1
Gastrojejunal anastomosis	2
Breakdown of complications	
Grade I (%)	59 (4.6%)
Grade II (%)	51 (4.0%)
Grade IIIa (%)	8 (0.6%)
Grade IIIb (%)	25 (2.0%)
Grade IV (%)	16 (1.3%)
Grade V (death) (%)	1 (0.1%)

Complications are ranked according to the Dindo-Clavien classification of postoperative complications

Results of the univariate and multivariate, regression analysis are shown in Table 3. Variables that were considered potential predictors of overall early complications on univariate analysis included age, BMI, sex, ASA score, T2D, hypertension, dyslipidemia, OSAS, GERD as well as the following laboratory parameters: plasma creatinine, albumin, ferritin and hemoglobin. Laboratory parameters were available only in the Geneva University Hospital cohort. Male sex and ASA score >2 (significant correlation with overall 30 day postoperative complications on univariate analysis), as well as Age >65, BMI>50 and T2D (clinically pertinent variables) were subsequently entered in a multivariate, logistical regression analysis. Male sex and ASA score >2 were identified as independent predictor of overall, 30 day postoperative complications (OR 1.680, 95% CI 1.172–2.409, *p*=0.005 and OR 1.673, 95% CI 1.172–2.389, *p*=0.005). None of the above mentioned variables was significantly correlated with severe (grade ≥IIIa) 30 day complications on either univariate or multivariate analysis.

**Table 3:** Univariate and multivariate regression analysis of predictors of overall, 30 day postoperative complications

Characteristics	Total number	Patients with complications	Univariate analysis			Multivariate analysis		
			OR	95% CI	*P*-value	OR	95% CI	*P*-value
Age, years								
<65	1219	153	1					
≥65	57	7	1.025	0.457–2.302	0.952	1.049	0.461–2.385	0.909
Sex								
Female	958	105	1					
Male	318	55	1.699	1.192–2.421	0.003*	1.680	1.172–2.409	0.005*
BMI, kg/m2								
<50	1040	125	1					
≥50	236	35	1.275	0.850–1.911	0.240	1.314	0.870–1.985	0.195
ASA								
≤2	555	53	1					
>2	721	107	1.651	1.164–2.342	0.005*	1.673	1.172–2.389	0.005*
T2D	442	53	0.926	0.651–1.316	0.667	0.908	0.633–1.303	0.601
Hypertension	561	59	0.857	0.584–1.257	0.428			
Dyslipidemia	923	122	1.263	0.858–1.859	0.237			
OSAS	432	55	1.201	0.823–1.754	0.342			
GERD	431	64	1.610	1.107–2.341	0.013*			
Creatinine^a^								
≤80 µmol/l	672	102	1					
>80 µmol/l	128	23	1.224	0.744–2.014	0.426			
Ferritin^a^								
≥30 µg/l	618	99	1					
<30 µg/l	147	16	0.640	0.365–1.123	0.120			
Albumin^a^								
≥35 g/l	647	100	1					
<35 g/l	128	18	0.689	0.521–1.539	0.689			
Hemoglobin^a^								
≥120 g/l	751	122	1					
<120 g/l	31	2	0.356	0.084–1.510	0.161			

*OR* odds ratio, *CI* confidence interval, *BMI* body mass index, *ASA* American society of anesthesiologists, *T2D* type 2 diabetes mellitus, *OSAS* obstructive sleep apnea syndrome, *GERD* gastroesophageal reflux disease

Asterisks indicate statistically significant *p*-values

^a^Data available only in the Geneva University Hospital cohort of patient

Given different baseline characteristics among patients from the USA and the Swiss cohort, subgroup univariate analyses by institution where performed for all primary and secondary outcomes and found no difference between subgroups.

## Discussion

This study found an overall 30 day complication rate of 12.5% (160/1276) and a severe 30 day complication rate of 3.9% (50/1276) after RRYGB. Male sex and ASA score >2 were identified as independent predictors of overall early postoperative complications on multivariate, logistical regression analysis. No predictor of severe early postoperative complications was identified among the analyzed variables. These findings are remarkably different compared to studies evaluating predictors of postoperative complications after LRYGB, which identified additional predictors such as high BMI or associated comorbidities (T2D, HTA, dyslipidemia) [9–11].

The low rates of early postoperative complications after primary RRYGB found in the present study are consistent with outcomes from other high-volume, accredited bariatric surgery centers [24–26]. Of note, the rates of severe complications (3.9%)—including a 0.2% (3/1276) rate of gastrointestinal leaks—are among the lower figures found in the literature, and confirm the conclusions of previous studies which showed robotically assisted bariatric surgery to be at least equivalent to standard laparoscopy in terms of safety [15–20]. Male sex has been previously identified as a risk factor for postoperative complications after bariatric surgery by several studies [9–11]. Most likely explanations include higher prevalence of excessive visceral fat and shorter mesentery lengths, which can sometimes increase the difficulty to perform RYGB in male patients [10, 27, 28]. As an established predictor of postoperative mortality, ASA score was expected to correlate with postoperative complications [29]. Interestingly, individual obesity-linked comorbidities were not identified as predictors in the present study, despite their contribution in defining patients ASA score. This finding suggest that overall patient health status and combination of several comorbidities are probably more predictive of postoperative complication after RRYGB than individual obesity-associated conditions. In addition, the fact that other traditional predictors such as BMI>50 were not correlated with poorer postoperative outcomes after RRYGB could suggest a potential benefit of the robotic approach for patients traditionally considered to be at higher risk of complications after LRYGB. The following elements could hypothetically explain this assumption. The increased ergonomics and dexterity offered by the robotic platform might help the surgeon overcome technical challenges seen in patients with super-obesity such as massive amounts of parietal and intra-abdominal fatty tissue. Furthermore, decreased postoperative complications rates have been described in patients undergoing robotically assisted revisional bariatric surgery [18–20]; this finding could also be true for other technically challenging bariatric procedures, such as primary RYGB in patients with super-obesity or multiple comorbidities. This hypothesis should however be taken with caution, since the present study did not include a comparative cohort of patients who underwent LRYGB.

The baseline differences seen between patients from the two institutions most likely reflect the different prevalence of obesity among the USA and the Swiss population. As expected [30], the mean BMI and the proportion of patients with BMI>50 were significantly higher in the US cohort, which predictably translates into higher ASA scores and increased prevalence of obesity-related comorbidities. Of note, the surprisingly lower prevalence of dyslipidemia seen in the US cohort (43.7% versus 88.8%, *p*<0.0001) is most likely due to more permissive plasma cholesterol and triglycerides cutoffs than those used in the Swiss cohort, in whom dyslipidemia was strictly defined according to the previously mentioned ASMBS guidelines [23].The authors were unfortunately unable to retrieve baseline plasma lipid panels to rectify the potentially underestimated prevalence of dyslipidemia among US patients. Diagnostic criteria of GERD were also different among cohorts. History of typical GERD symptoms were used in Swiss patients, versus the GerdQ questionnaire in US patients, potentially underestimating the prevalence of GERD in the former cohort due to suboptimal sensitivity [31]. Given this potential bias and the lack of clinical pertinence, GERD was not included in the multivariate regression analysis despite being identified as a potential predictor on univariate analysis.

This study has several limitations. As a registry-based study, it is retrospective in nature and subject to collecting biases, which the authors aimed to reduce by manually verifying each patient record to ensure data accuracy and completeness. Patient populations were also significantly different between the two centers, which could have led to potential biases in the joint data analysis. To minimize this risk, subgroup analyses by center were therefore initially performed and found no difference in primary and secondary outcomes between the two cohorts. As mentioned in the previous paragraph, different diagnostic criteria were used for USA and Swiss patients to define dyslipidemia and GERD, which could have masked potential correlations with early postoperative complications. The lack of laboratory data in the US cohort of patients might have also resulted in insufficient statistical power to detect a potential predictor among the analyzed blood parameters. Comparisons between the laparoscopic and the robotic approach should be considered with caution due to the absence of a comparative cohort of patients undergoing LRYGB.

In conclusion, this multicenter study identified male sex and ASA >2 as independent predictors of overall early postoperative complications after primary RRYGB. No predictor of severe postoperative complications were identified. These findings suggest a potentially different risk profile after RRYGB compared to LRYGB. The smaller number of predictors combined with low rates of postoperative complications could suggest a potential benefit of RRYGB in patients with previously published predictors of increased morbidity after LRYGB, such as patients with BMI>50 or obesity-related comorbidities, with the caveat that the present study was not designed to assess a difference between the two techniques. Larger, prospective studies are necessary to confirm these results.
